# Adrenocorticotropic Hormone Producing Pituitary Carcinoma in the Falx Cerebri, Retroclival Region, Ethmoidal Cells, and Other Locations

**DOI:** 10.7759/cureus.63735

**Published:** 2024-07-03

**Authors:** Jose E Esquivel, Ana B Santos, Anthony Hong, Alejandro Cob Guillén

**Affiliations:** 1 Endocrinology, Hospital San Juan de Dios, San José, CRI; 2 Medicine, University of Costa Rica, San José, CRI

**Keywords:** falx cerebri, pituitary macroadenoma, cushing disease, pituitary carcinoma, adrenocorticotropic hormone

## Abstract

Pituitary carcinoma is a condition defined by metastasis of a pituitary tumor to a distant location, and it is a very rare type of adenohypophyseal tumor. We present a case of a 29-year-old female who was followed up in our Endocrinology Department. Past medical history included the diagnosis of Cushing disease and transsphenoidal tumor resection at 12 years of age, followed by transcranial resection two years later because of persistently elevated adrenocorticotropic hormone (ACTH). Despite the surgical management, the patient persisted with increased ACTH and hypercortisolism, and, thus, bilateral adrenalectomy was performed a year later. Two years after the procedure, the patient presented with a newly diagnosed pituitary macroadenoma, and the diagnosis of Nelson syndrome was made. Linear accelerator radiotherapy was given, which reduced the size of the tumor. Later, several imaging studies showed multiple lesions on the falx cerebri, posterior clinoid process, retroclival region, cerebellopontine angle, pterygopalatine fossa, infratentorial region, and posterior ethmoidal cells. Biopsy and immunohistochemistry of the falx cerebri lesions described ACTH-producing pituitary adenocarcinoma. Treatment was given with intramuscular octreotide, dabrafenib, and trametinib. Despite persistently elevated ACTH levels, the patient has since remained clinically stable, without new development or worsening of symptoms. There are three unique aspects of our case. First, we reported an unusual presentation of this disease, since the patient in our case was a female with an early age of onset. Second, this is the first reported case demonstrating pituitary carcinoma in the falx cerebri. Third, the prognosis of pituitary carcinoma is usually very poor, and mortality is extremely high; however, the patient in our case has been followed up for seven years since the diagnosis of the metastatic lesions and has remained clinically stable.

## Introduction

Pituitary adenomas are common, benign tumors that arise from the endocrine cells of the anterior pituitary gland and require a multidisciplinary treatment of local mass effects and peripheral endocrinopathies [1]. In some cases, pituitary tumors can present an aggressive behavior and can metastasize. Those tumors of adenohypophyseal cells exhibiting metastasis are called pituitary carcinomas (PCs) [2]. PCs are very rare and account for approximately 0.12% of adenohypophyseal tumors [3]. They usually originate from functioning tumors with the most common ones being corticotroph and lactotroph carcinomas [2]. Corticotroph PCs typically occur in three settings: corticotroph tumors that secrete adrenocorticotropic hormone (ACTH), corticotroph carcinoma that present after a bilateral adrenalectomy, and “silent” corticotroph tumors where corticotroph tumors secrete precursors of ACTH that may be measurable in the circulation but cannot easily bind and activate the ACTH receptor [4]. We present the case of a patient with a history of refractory Cushing disease, who was diagnosed with a corticotroph PC in the falx cerebri and other unusual locations.

## Case presentation

A 29-year-old female with a history of Cushing disease was followed up by our department because of persistently increased ACTH levels. Past medical history was relevant for a transsphenoidal resection of an ACTH-producing pituitary adenoma at 12 years of age, followed by a transcranial resection two years later. Despite these surgeries, the patient persisted with increased ACTH and hypercortisolism, but a head CT scan at 15 years of age showed no recurrence of the tumor, and, thus, bilateral adrenalectomy was performed.

The patient developed progressively skin hyperpigmented lesions and had increased ACTH. A head magnetic resonance imaging (MRI) at 17 years of age showed a new pituitary macroadenoma measuring 15x2.5mm, and another head MRI performed a year later showed increased size of the lesion, measuring 20x9x14mm, with suprasellar extension and thus compression of the right cavernous sinus and optic chiasm. The diagnosis of Nelson syndrome was made because of these findings. Linear accelerator radiotherapy was given, which reduced the pituitary adenoma to 9x6mm in a follow-up MRI, but it also showed several isolated lesions in the falx cerebri and right posterior clinoid process with suggestive characteristics of meningiomas.

The patient continued receiving outpatient care, but because ACTH levels progressively increased, another MRI was performed at 23 years of age, which showed an increased number of lesions in the falx cerebri. A biopsy of one of these lesions showed round-nucleated homogenous cells with pink cytoplasm. Immunohistochemistry staining for synaptophysin, ACTH, and S-100 was positive, and Ki67 index was 3%. This corresponded to an ACTH-producing PC.

**Figure 1:**
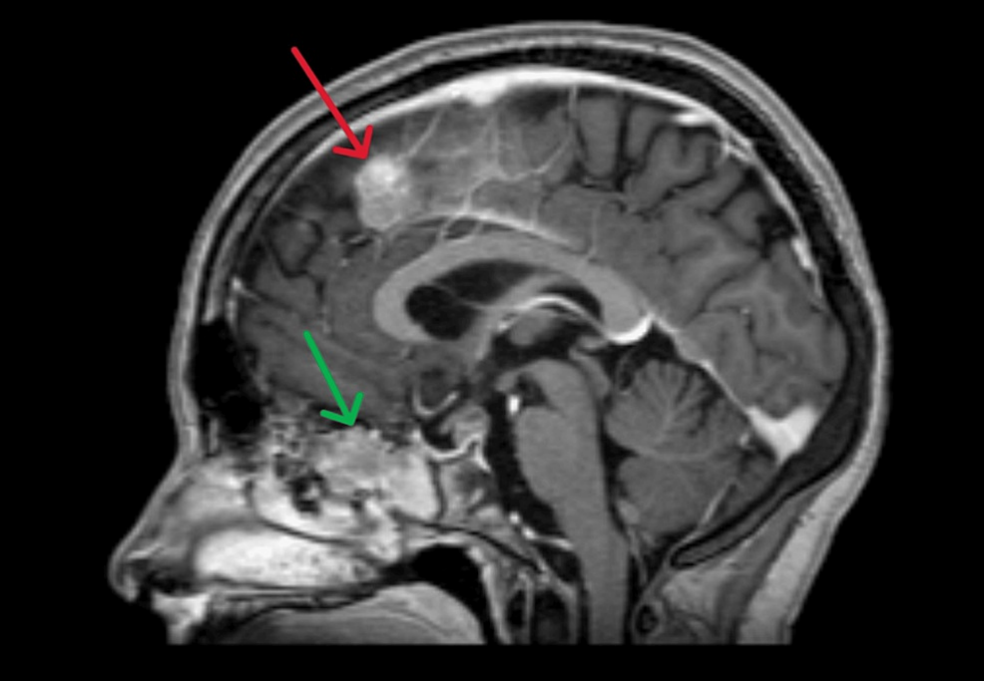
Sagittal T1 MRI with contrast. The red arrow indicates a lesion in a left parasagittal position of the falx cerebri. The green arrow indicates the lesion located in the posterior ethmoidal cells.

**Figure 2:**
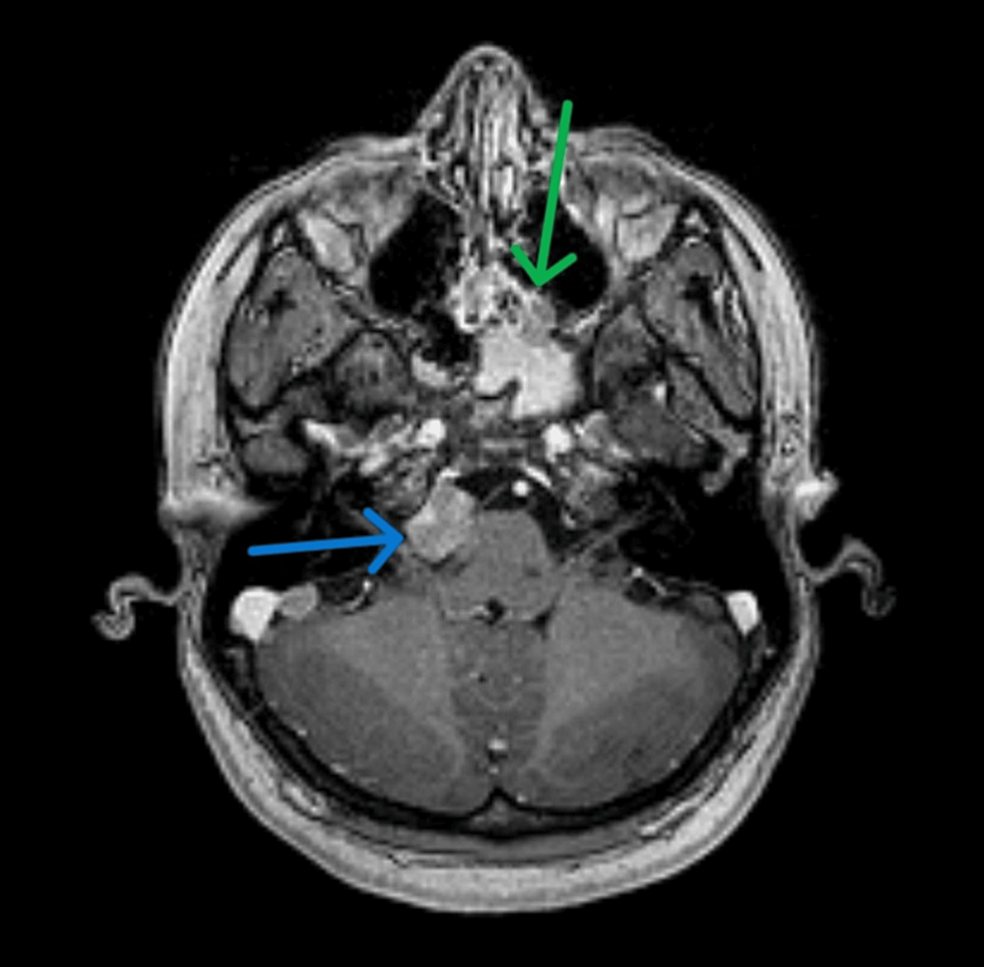
Axial T1 MRI with contrast. The green arrow indicates the lesion in the posterior ethmoidal cells. The blue arrow indicates a lesion in the right cerebellopontine angle causing slight compression of the pons.

An Octreoscan was performed at 25 years of age, which showed at least three focal lesions with increased captation: one in the anterior falx cerebri, one projected in the quadrigeminal cistern, and one in the right cerebellopontine angle. No other lesions were found in a full-body scan, and the patient only received medical treatment. At 28 years of age, an MRI was performed (Figure 1), which showed two lesions in the parasagittal region of the falx cerebri (one 8mm lesion on the right and a 14x6mm lesion on the left). Additional lesions were also noted (Figure 2): a 17x12mm lesion in the right retroclival region, a 16x14x15mm in the left retroclival region with associated hematoma, a 10mm lesion posterior to the right cerebellopontine angle, an 8mm lesion towards the right side of the foramen magnum, and an anterior one of 4mm, an 8mm lesion in the left pterygopalatine fossa, a 10mm lesion in the right posterior infratentorial region, and a 13x23x19mm nodule in the posterior ethmoidal cells.

**Figure 3:**
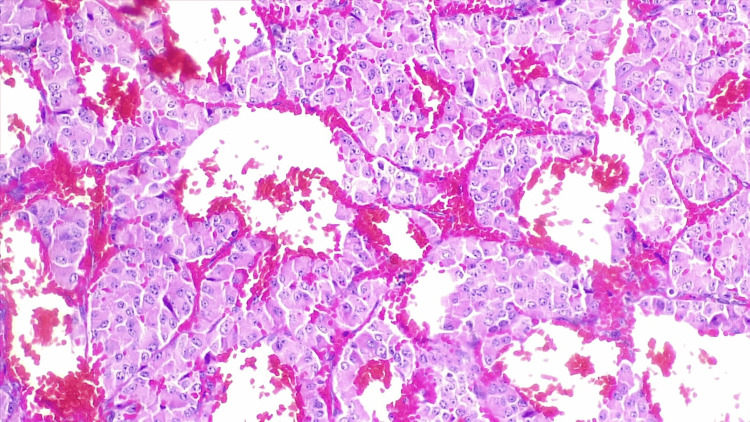
A biopsy of a falx cerebri lesion showing round-nucleated homogenous cells with pink cytoplasm.

Another biopsy of one of the lesions was taken (Figure 3), which showed the same histologic findings as the previous one, with a mutation in BRAF V600E, which confirmed the diagnosis of ACTH-producing PC.

The patient did not receive more surgical management. Instead, outpatient management consisted of 40mg of intramuscular octreotide administered monthly. After the most recent biopsy findings, she has been treated with dabrafenib (150mg twice a day, taken orally) and trametinib (2mg per day, taken orally). Her last measured serum cortisol and potassium levels were 6.1µg/dL (reference values: 6.02-18.4µg/dL) and 4.1mEq/L (reference values: 3.5-5.0mEq/L), respectively. The patient has received outpatient management until the present date and has remained clinically stable, without worsening of her ACTH-related symptoms or developing new symptoms, although her last ACTH level was >2,000pg/mL.

## Discussion

PC is a rare condition defined by metastasis of a pituitary tumor to a distant location [5]. Although PC can present at any age, it typically presents in the third to fifth decade of life (median of 58 years) and has a male predominance (62.2%) [3]. However, we reported an unusual presentation of this disease, because the patient in our case was a female with an early age of onset. The diagnosis of PC requires a high suspicion, radiographic imaging with detection of metastases, and a pathologic confirmation of pituitary origin of the metastases.

PC can present many years after the diagnosis of a pituitary adenoma and should be suspected in the case of discordant biochemical and radiological findings, or when a patient with a previous diagnosis of a locally invasive pituitary adenoma is discovered to have lesions in the brain or at extracranial sites that suggest metastasis [2,6]. In our case, corticotroph PC could be suspected when the patient presented with elevated levels of ACTH despite the surgical resection of the pituitary adenoma. In the diagnosis workup of PC, in addition to MRI, an Octreoscan can be performed to characterize the metastasis. Radioscintigraphy with I-111 octreotide has been reported to aid in the diagnosis of metastatic ACTH-secreting carcinomas, although its sensitivity has not been well established [6].

PC is confirmed with a biopsy of suspected metastasis and immunochemical staining. However, cytologic features of PC can be similar or indistinguishable from adenomas [7]. Moreover, no immunohistochemical markers have been identified that conclusively distinguish pituitary adenoma from a PC [8]. Ki-67 has been used as a potential prognostic pathological marker of PC when the mitotic index is >3% [2].

Currently, given the rarity of PCs, there is no definitive consensus or standardized treatment for them. Treatment modalities include surgical resection for the primary pituitary mass, medical therapy, radiation therapy, and chemotherapy [7].

Because PCs are diagnosed only when tumor dissemination has occurred, the prognosis is very poor and mortality is extremely high [8]. However, the patient in our case has been followed up for seven years since the diagnosis of the metastatic lesions and has remained clinically stable, with no life-threatening conditions. Therefore, efforts should be focused on early diagnosis and treatment.

## Conclusions

PC is a rare condition defined by metastasis of a pituitary tumor to a distant location. We describe a case with an unusual presentation of this disease because the patient in our case was a female with an early age of onset, in contrast to the reported literature. Also, this is the first reported case demonstrating PC in the falx cerebri. The prognosis of PC is very poor, and mortality is extremely high; however, the patient in our case has been followed up for seven years since the diagnosis of the metastatic lesions and has remained clinically stable. Clinicians should be aware that this disease has a bad prognosis and can present many years after the diagnosis of a pituitary adenoma and should be suspected in the case of discordant biochemical and radiological findings.
